# Personalized Transcranial Direct Current Stimulation for Behavioral and Neurophysiologic Outcomes

**DOI:** 10.1001/jamanetworkopen.2025.26148

**Published:** 2025-08-25

**Authors:** Sagarika Bhattacharjee, Palanimuthu T. Sivakumar, Ganesan Venkatasubramanian, Rose Dawn Bharath, Kenichi Oishi, Brenda Rapp, John E. Desmond, S. H. Annabel Chen, T. N. Sathyaprabha, Kaviraja Udupa, Rajan Kashyap

**Affiliations:** 1 Department of Neurophysiology, National Institute of Mental Health and Neurosciences, Bengaluru, India; 2 Department of Neuroimaging and Interventional Radiology, National Institute of Mental Health and Neurosciences, Bengaluru, India; 3 Department of Psychiatry, National Institute of Mental Health and Neurosciences, Bengaluru, India; 4 Department of Neurology, Johns Hopkins University School of Medicine, Baltimore, Maryland; 5 Department of Cognitive Science, Johns Hopkins University, Baltimore, Maryland; 6 Department of Psychology, School of Social Sciences, Nanyang Technological University, Singapore; 7 Lee Kong Chian School of Medicine, Nanyang Technological University, Singapore; 8 Centre for Research and Development in Learning, Nanyang Technological University, Singapore

## Abstract

**Importance:**

Transcranial direct current stimulation (tDCS) is emerging as a home-based intervention for neuropsychiatric conditions and cognitive enhancement. However, its effectiveness is limited by interindividual variability, as fixed-dose protocols have failed to account for anatomic differences influencing current delivery to targeted regions and treatment outcome. While computational modeling supports individualized dosing to improve consistency, experimental validation remains limited.

**Objective:**

To compare the behavioral and neurophysiologic outcomes of fixed-dose vs individualized-dose tDCS.

**Design, Setting, and Participants:**

This comparative effectiveness study using a within-participant, double-masked, crossover design was conducted from January 1, to March 31, 2024, at the National Institute of Mental Health and Neurosciences in India. Adult participants (aged 21-35 years) received 3 sessions of tDCS (fixed-dose, individualized-dose, and sham stimulation) in counterbalanced order. Individualized doses were calculated using a custom-built simulation toolbox.

**Main Outcomes and Measures:**

Behavioral performance was measured using reaction time during a rapid naming task. Neurophysiologic effects were assessed using motor-evoked potentials (MEPs) recorded before and after stimulation. Linear mixed-effects models were used for the statistical analysis.

**Results:**

Sixteen right-hand-dominant, bilingual English-Dravidian speakers (mean [SD] age, 23.1 [3.9] years; 8 female [50%]) were included. Individualized-dose tDCS associated with significantly greater reaction time improvement over sham (estimated marginal mean [SD]: before, 753.0 [41.1] ms; after, 619.0 [41.1] ms; change [Δ] = 133.6 ms; SE, 10.2 ms; *z* score ratio, 13.09) compared with fixed-dose tDCS (before, 694.0 [41.1] ms; after, 680.0 [41.1] ms; Δ = 14.6 ms; SE, 10.1 ms; *z* score ratio, 1.45). Variability was lower with individualized-dose stimulation (coefficient of variation, −1.14 vs 0.39 fixed vs individualized dose, respectively). Sex-stratified analyses showed that women had improvements with both fixed (Δ = 58.0 ms; *P* = .003) and individualized (Δ = 113.8 ms; *P* < .001) stimulation, while men had improvement only with individualized tDCS (Δ = 153.4 ms; *P* < .001). Seven participants (5 men and 2 women) converted from nonresponders to responders with individualized dosing. For MEPs, individualized-dose tDCS showed greater poststimulation amplitude increases over sham (β [SE], 0.91 [0.23]), although fixed-dose tDCS poststimulation amplitude was smaller, but significant (β [SE], 0.56 [0.23]; *P* = .02) and showed a higher percentage change (β [SE], 144.26% [55.74%]; *P* = .01) and reduced variability (coefficient of variation, −0.79 vs 1.12 [fixed]).

**Conclusions and Relevance:**

In this comparative effectiveness study, dose-controlled tDCS was associated with consistent behavioral and neurophysiologic improvement, highlighting its translational importance in the treatment of neuropsychiatric disorders.

## Key Points

**Question:**

What is the association of personalizing transcranial direct current stimulation (tDCS) dosing with behavioral and neurophysiologic therapy precision?

**Findings:**

In this comparative effectiveness study in 16 participants, individualized tDCS was associated with significantly greater improvements than fixed-dose tDCS over sham in behavioral and neurophysiologic outcomes and with reduced interindividual variability. Several participants unresponsive to fixed-dose tDCS responded with personalized dosing, with no sex differences observed.

**Meaning:**

These findings suggest that individualized tDCS may enhance efficacy, reduce sex-related variability, and improve responsiveness, supporting its potential for broader clinical use.

## Teaser

This comparative effectiveness study compares the behavioral and neurophysiologic outcomes associated with fixed-dose vs individualized-dose transcranial direct current (tDCS) stimulation.

## Introduction

The use of transcranial direct current stimulation (tDCS) as a home-based intervention for neuropsychiatric disorders is rapidly gaining momentum, driven by its noninvasive nature, minimal adverse effects, affordability, and ease of use.^1,2^ Traditionally, tDCS protocols have relied on fixed electrode placements (called montages) and current doses. While such settings modulate cortical excitability, their effects vary widely due to individual anatomic differences that influence the amount of current reaching the target region.^3^ In fact, fixed-dose tDCS can result in up to 100% variability in current density at the target site.^4^ To improve precision, electric field models have been developed to estimate current distribution in an individual’s brain based on montage and dose parameters.^5^ Computational modeling suggests that individualized dosing might eliminate this variability and enhance efficacy,^6^ although experimental validation is lacking. This study addresses the gap in experimental validation of individualized tDCS dosing by conducting a within-participant experiment in which participants received both fixed and individualized doses targeting the left inferior parietal lobule (IPL), a key region in the language network involved in phonologic and semantic processing.^7,8^ The study compares the outcomes of these dosing conditions for language performance and cortical excitability, providing crucial empirical insights into the potential benefits of personalized tDCS dosing.

## Methods

### Participants

This comparative effectiveness study included healthy, right-hand-dominant adults aged 21 to 35 years who were native English speakers with normal or corrected-to-normal vision and no prior exposure to tDCS. The study was approved by the institutional ethics committee of the National Institute of Mental Health and Neurosciences, and all participants provided written informed consent. The study followed the International Society for Pharmacoeconomics and Outcomes Research (ISPOR) reporting guideline.

Participants were recruited via institutional advertisements and word of mouth. All participants met standard magnetic resonance imaging (MRI) and transcranial magnetic stimulation (TMS) safety criteria. Exclusion criteria included a history of neurologic, psychiatric, or reading disorders; head trauma; epilepsy; psychiatric medication use; pregnancy or breastfeeding; and contraindications, such as metal implants, pacemakers, claustrophobia, seizures, or dermatologic conditions at stimulation or recording sites.

Consistent with prior tDCS studies showing robust effect sizes for behavioral and neurophysiologic outcomes for crossover study design, the sample size was capped at 16 participants.^9,10,11^ Specifically, prior work from our laboratory reported Cohen *d* values ranging from 0.68 to 0.88 in tDCS-induced modulation of reaction time in a bilingual reading task.^11^ Power calculations based on these estimates indicated that a sample of 16 participants would provide approximately 75% to 80% power to detect meaningful within-participant outcomes, making this design suitable for effect estimation and hypothesis generation.

### Experiment Design

**Figure 1.**
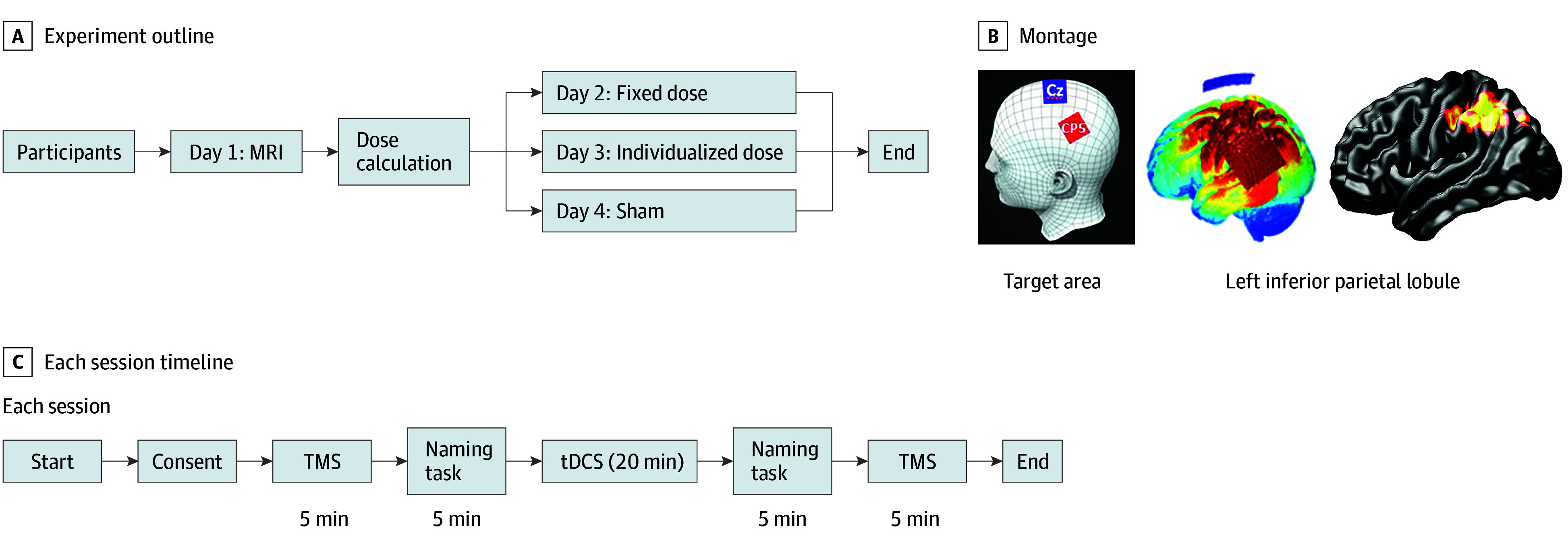
Experimental Workflow and Stimulation Protocol A, Participants underwent magnetic resonance imaging (MRI) to enable individualized transcranial direct current stimulation (tDCS) dose calculation using electric field modeling. Each participant then completed 3 double-masked, counterbalanced sessions receiving fixed-dose, individualized-dose, and sham tDCS. B, Stimulation was administered using a CP5-CZ electrode montage targeting the left-side inferior parietal lobule, as confirmed by simulation of current density. C, Each session included a standardized timeline consisting of informed consent, prestimulation motor-evoked potential recording via transcranial magnetic stimulation (TMS), a rapid naming task, 20 minutes of tDCS, and poststimulation naming task and TMS assessments.

A within-participant, double-masked, comparative crossover study was conducted from January 1 to March 31, 2024, at the National Institute of Mental Health and Neurosciences in India. The experimental protocol is illustrated in Figure 1. High-resolution T1-weighted brain images were acquired for each participant using an Ingenia 3.0T MRI scanner (Koninklijke Philips). These images were processed using the Individualized Systematic Approach for tDCS Analysis (i-SATA), a built-in, widely used, open-source toolbox, to determine individualized tDCS dosage.^12^ The personalized dosing approach adjusted the applied current to ensure that all participants received the same amount of current density at the target region. The i-SATA toolbox has been theoretically validated across small^11^ and large datasets^13,14,15^ and experimentally used in studies of behavior alone^11,16^ and with multiple modalities (electroencephalography and online tDCS).^17^ Its application in multiple studies has highlighted its importance in estimating current density at an individual’s target region.

Each participant underwent 3 randomized, double-masked tDCS sessions (2-mA fixed-dose, individualized-dose, or sham), spaced at least 24 hours apart to minimize carryover effects. Stimulation order was randomized and counterbalanced using a Latin square design by an independent researcher. Masking was ensured via preencoded protocols. The 24-hour time gap was chosen based on previous evidence showing that tDCS aftereffects in healthy adults are short-lived, typically resolving within 1 to 2 hours.^18,19^ Sessions were held at the same time of day to control for circadian influences,^20^ with consistent environmental conditions. Participants avoided alcohol, caffeine, and strenuous activity before sessions.

Stimulation was delivered for 20 minutes at CP5 (anode) and CZ (cathode) using a 5 × 5 cm^2^ montage, targeting the left IPL, a region optimally stimulated by this montage^21^ and commonly used in language-task studies.^17^ Sham stimulation included a 30-second ramp-up and ramp-down to mimic the sensation of active tDCS. Pre- and poststimulation assessments included measurement of reaction times from rapid naming tasks and TMS-induced motor-evoked potentials (MEPs) to evaluate behavioral and physiologic outcomes of tDCS, respectively. The outcomes of this cortical excitation were quantified at the first dorsal interosseous through measurement of MEP amplitude and used as a marker of the neurophysiologic effects of tDCS. Side effects and masking assessments were also recorded to ensure participant safety and study integrity.

### MRI Scanning

High-resolution, 3-dimensional, T1-weighted images were acquired using a fast field echo sequence (repetition time, 6.5 ms; echo time, 2.9 ms; flip angle, 9°; voxel size, 1 × 1 × 1 mm^3^; field of view, 256 × 256 × 211 mm^3^; matrix, 256 × 256; 211 sagittal slices; turbo field echo factor, 256). Parallel imaging (sensitivity encoding) was applied (anterior-posterior, 1; right-left, 2). No fat or water suppression was used. Total scan time was approximately 6 minutes; safety parameters remained within normal limits.

### Individualized Dose Calculation

The procedure to calculate the individualized dose for each participant has been described in detail in Bhattacharjee et al.^22^ For each individual’s MRI, electric field simulations were performed using Realistic, Volumetric Approach to Simulate Transcranial (ROAST), version 3.0 open-source software^23^ for the following montage configuration: (1) anode at CP5, cathode at CZ; (2) electrode size 5 × 5 cm^2^; and (3) default conductivity values (as set for each tissue layer in ROAST) for white matter (0.126 S/m), gray matter (0.276 S/m), cerebrospinal fluid (1.65 S/m), bone (0.01 S/m), skin (0.465 S/m), air (2.5 × 10^−14^ S/m), gel (0.3 S/m), and electrode (5.9 × 10^7^ S/m).^24,25,26^ The current dose was fixed at 2 mA (fixed dose), an arbitrary choice based on the majority of the tDCS studies that used this value in their experiment.^27,28,29,30^ The simulations provided current density values for all x, y, and z brain coordinates (Figure 1B). The i-SATA software mapped the cortical locations of the output of ROAST to the automated anatomic labeling atlas with 116 regions^31^ and calculated the average current density at the left-side IPL (target region).^12,13,14,16,32^ For an individual, the intensity obtained at the target region of interest (ROI) is referred to as simulated intensity (*M*_simulated − intensity_).

To calculate the individualized dose, the current density at the target region was also calculated for the standard Montreal Neurological Institute coordinate system brain with the aforementioned prespecified montage setting.^21^ The current density obtained at the target ROI using a fixed dose of 2 mA was set as the reference intensity (*M*_reference − intensity_). For every individual, the intensity at the target ROI needs to be equal to *M*_reference − intensity_; thus, the amount of current dose required by each individual to achieve the reference current density at the target ROI is the individualized dose. This dose can be obtained using the following equation^6^: individualized dose = (*M*_reference − intensity_ / *M*_simulated − intensity_) × fixed dose, where fixed dose = 2 mA.

### Outcome Measures and Analysis

The rapid naming task lists were previously validated in a bilingual population^11^ and reaction times were measured. The MEPs were recorded using a well-calibrated MagVenture TMS system (MagVenture Inc), following international guidelines.^33^ Although MEP amplitude is a widely used marker of corticospinal excitability, it is known to exhibit inherent variability,^34,35^ which was addressed through rigorous trial averaging and standardized stimulation parameters.

#### Behavioral Task Conducted Before and After Stimulation

The rapid naming task was selected due to its sensitivity to neuromodulation of language-related regions, particularly the left-side IPL. As a time-sensitive measure of lexical access and phonologic retrieval, it served as an appropriate index to compare individualized and fixed tDCS dosing effects. Six equivalent 50-word English lists were created from 300 words sourced from psycholinguistic corpora, balanced for frequency, length, phonologic and orthographic neighbors, and age of acquisition. A 1-way analysis of variance test confirmed no significant differences between lists (*P* > .05). Rapid naming tasks were presented via E-Prime (Psychology Software Tools), with each word shown for 2000 ms. Reaction times were recorded using a voice key and audio, and accuracy was scored based on pronunciation norms in British, US, and colloquial Indian English. Task details, reliability, and validity are described in prior work.^11^

#### MEP Recording and Preprocessing

During the first visit, participants completed a structured medical history, handedness assessment (Edinburgh Handedness Inventory), and safety screening. For the experiment, they sat comfortably with arms at 90°, remaining quiet and alert during TMS. Electromyography (EMG) was recorded from the dominant first dorsal interosseous using PowerLab 4/25-T (ADInstruments), with electrodes on the muscle belly, second finger, and ipsilateral ulnar styloid. Signals were digitized at 1 kHz (±10 mV, 0.3- to 1000-Hz filter). Transcranial magnetic stimulation was delivered via a MagPro X100 and Cool-B65 coil (MagVenture Inc) over the motor cortex at a 45° angle. The motor hotspot was identified to determine resting motor threshold, defined as the lowest intensity evoking at least 50-µV MEPs in 50% of trials. Baseline corticomotor reactivity was assessed with 40 MEPs at 120% resting motor threshold. Postintervention MEPs were analyzed as percentage change from baseline in sets of 10. Cortical silent period was measured using 10 pulses at 120% resting motor threshold during 25% isometric first dorsal interosseous contraction with EMG feedback. Cortical silent period duration was averaged after excluding outliers (>2.5 SDs), with 15 to 20 valid trials per participant, following the procedure described by Nuyts et al.^36^ Bad trials were excluded by visual inspection. Trials were classified as bad if they exhibited excessive noise, prestimulus muscle activation, motion artifacts, or electrode instability or lacked a clearly discernible MEP response. The MEP amplitude, latency, and cortical silent period were computed using the signal package in R, version 4.5.1 (R Foundation for Statistical Computing).

### Statistical Analysis

Linear mixed-effects (LMEs) models were used to examine the associations of stimulation condition (fixed dose, individualized dose, and sham), time (before vs after), and sex with behavioral (reaction time) and neurophysiologic (MEP amplitude) outcomes. All models were implemented using the lmer function from the lme4 package in R, with statistical significance assessed using Satterthwaite approximation for degrees of freedom (via lmerTest), and post hoc contrasts estimated using the emmeans package, adjusted for multiple comparisons using the Tukey method.

#### Association of tDCS With Reaction Time

##### Main Model

A primary LME model was constructed for reaction time that included fixed effects of tDCS type (fixed dose, individualized dose, and sham), time (before and after), sex, and their interactions. Random intercepts were included for participant and trial numbers to account for repeated measurements. Post hoc tests examined interventional (before and after) changes within each tDCS type.

##### Subgroup Comparison of Fixed vs Individualized Dose

A secondary LME analysis was performed on a subset of data limited to the fixed-dose and individualized-dose tDCS conditions. This analysis allowed direct testing of interactions (tDCS type × time × sex) and estimation of condition-wise contrasts.

##### Proportional Change From Baseline Performance

To quantify individual responsiveness, percentage change scores were calculated at the trial level as ([after − before] / before) × 100. The percentage change values were analyzed using LMEs, with tDCS and sex as fixed factors and participant and trial numbers as random factors. Post hoc contrasts estimated the percentage change in differences between the 2 groups (fixed dose and individualized dose).

##### Brain-Behavior Association

The association between the current density and reaction time was examined using a linear regression performed in the fixed-dose condition with percentage reaction time change estimated by current density at the left-side IPL and sex and their interactions. In the individualized-dose condition, direct sex-based comparisons were performed, since current density was constant across individuals (a criteria used to individualize the dose).

#### Association of tDCS With MEP

All statistical analyses conducted for reaction time were applied to MEP amplitude. This analysis was used to quantify the stimulation effects across both behavioral and neurophysiologic outcomes.

#### Responder and Nonresponder Analysis

To classify responders vs nonresponders, participant-wise change in reaction time and MEP (as a percentage) was computed before and after stimulation. Participants showing a negative change in reaction time and a positive change in MEP were classified as responders; others were classified as nonresponders. This binary classification, valid for active conditions (fixed and individualized dose), allowed additional analyses on a subgroup of individuals whose fixed and individualized doses were the same.

#### Reliability Analysis and Reporting Standards

In experiments related to precision in a crossover design, consistency of a measure or test when administered to the same individuals at different times needs to be evaluated. Test-retest reliability was assessed using intraclass correlation coefficients (ICCs), specifically the value ICC(A,1), which reflects absolute agreement of single measurements based on a 2-way random-effects model, appropriate for evaluating the consistency of individual responses across sessions. A 2-way random-effects model estimates test-retest reliability by accounting for variability across both participants and measurement sessions, treating both as random samples. Moreover, to evaluate the potential influence of testing day, session day was included as a covariate in an additional LME analysis, and both main effects and interactions of the tDCS condition on behavioral (reaction time) and neurophysiologic (MEP) outcomes were estimated.

Additionally, post hoc power analyses were conducted for the LME models using the simr package in R. Power was estimated running the fixed effects (tDCS and time) with 1000 permutations. The models were specified to report interaction effects at an α of .05.

In accordance with the ISPOR Good Research Practices for Comparative Effectiveness Research, we explicitly defined the intervention conditions, used a within-participant crossover design to minimize selection bias, and applied appropriate statistical adjustments (LMEs with multiple comparisons) to account for individual- and trial-level variability. Outcome measures, subgroup analyses, and effect size estimates are also reported in line with ISPOR’s recommendations for transparent interpretation and reproducibility of results.^37^

## Results

Sixteen right-hand-dominant, bilingual English-Dravidian speakers (mean [SD] age, 23.1 [3.9] years; 8 female [50.0%] and 8 male [50.0%]) were selected based on English proficiency. All participants tolerated tDCS well, with tingling being the most common adverse effect (fixed dose, 14 participants [87.5%]; individualized dose, 16 participants [100%]; sham, 13 participants [81.3%]), followed by mild itching, burning, and sleepiness. The nonsignificant χ^2^ test result (χ^2^ = 1.73; *P* = .42) suggested that participant’s guesses about the stimulation condition were no better than random, confirming effective masking.

### Associations of tDCS With Reaction Time

#### Overall Model

An LME model was used to assess the associations of tDCS condition (fixed dose, individualized dose, sham), time (before vs after), and sex with reaction time, with random intercepts for participant and trial numbers (Table). The model revealed significant main effect sizes of tDCS for fixed dose (β [SE], 32.41 [14.43]; *t*_4775_ = 2.24; *P* = .03), individualized dose (β [SE], 46.89 [14.43]; *t*_4775_ = 3.24; *P* = .001), time (β [SE], 57.98 [14.43]; *t*_4775_ = 4.01; *P* < .001), and several key interactions. A robust tDCS-by-time interaction was observed for both the fixed-dose (β [SE], −79.65 [20.15]; *t*_4775_ = −3.95; *P* < .001) and individualized-dose (β [SE], −171.81 [20.40]; *t*_4775_ = −8.41; *P* < .001) conditions, indicating significantly greater reaction time reductions over time, especially with individualized stimulation. Additionally, a significant tDCS-by-sex interaction (β [SE], −91.36 [20.40]; *t*_4775_ = −4.47; *P* < .001) and time-by-sex interaction (β [SE], −83.45 [20.40]; *t*_4775_ = −4.08; *P* < .001) suggested sex-specific modulation, further supported by a 3-way interaction for the fixed condition (tDCS type × time × sex: β [SE], 97.55 [28.68]; *t*_4775_ = 3.40; *P* < .001).

**Table.** Summary of LME Analyses Examining the Association of tDCS Condition, Time, and Sex With Behavioral (Reaction Time) and Neurophysiologic (MEP Amplitude) Outcomes

LME model	Estimate, β (SE)	*df*	*t*	*P *value
**Reaction time estimate: tDCS type × time × sex + participant No. + trial No.^a^**				
Fixed effects				
Intercept	635.36 (57.24)	16	11.1	<.001
tDCS (fixed dose vs sham)	32.41 (14.43)	4775	2.24	.03
tDCS (individual dose vs sham)	46.89 (14.43)	4775	3.24	.001
Time	57.98 (14.43)	4775	4.01	<.001
Sex	144.58 (79.70)	15	1.81	.09
tDCS (fixed dose vs sham) × time	−79.65 (20.15)	4775	−3.95	<.001
tDCS (individualized dose vs sham) × time	−171.81 (20.40)	4775	−8.41	<.001
tDCS (fixed dose vs sham) × sex	−91.36 (20.40)	4775	−4.47	<.001
tDCS (individualized dose vs sham) × sex	−3.67 (20.40)	4775	−0.18	.85
Time × sex	−83.45 (20.40)	4775	−4.08	<.001
tDCS (fixed dose vs sham) × time × sex	97.55 (28.68)	4775	3.40	<.001
tDCS (individualized dose vs sham) × time × sex	43.88 (28.86)	4775	1.52	.12
**Reaction time estimate: tDCS type × time × sex + participant No. + trial No. (only fixed vs individualized dose)^b^**				
Fixed effects				
Intercept	667.77 (50.22)	16	13.29	<.001
tDCS (individualized dose)	14.48 (14.84)	3179	0.97	.32
Time after stimulation	−22.04 (14.49)	3180	−1.52	.12
Male sex	53.22 (69.67)	15	0.76	.45
tDCS (individualized dose) × time after stimulation	−91.78 (20.74)	3179	−4.42	<.001
tDCS (individualized dose) × male sex	87.69 (20.98)	3179	4.17	<.001
Time after stimulation × male sex	14.48 (20.74)	3179	0.69	.48
tDCS (individualized dose) × time after stimulation × male sex	−54.06 (29.50)	3179	−1.83	.06
**Percentage reaction time change estimate: tDCS × sex + participant No. + trial No.^c^**				
Fixed effects				
Intercept	10.52 (5.10)	16.06	2.06	.05
tDCS (fixed dose)	−11.43 (2.28)	2374	−5.01	<.001
tDCS (individualized dose)	−23.55 (2.28)	2374	−10.32	<.001
Male sex	−10.64 (7.22)	16	−1.47	.16
tDCS (fixed dose) × male sex	14.60 (3.22)	2374	4.52	<.001
tDCS (individualized dose) × male sex	12.36 (3.22)	2374	3.83	<.001
**Amplitude estimate: tDCS type × time × sex + participant No. + trial No.**				
Fixed effects				
Intercept	4.11 (0.24)	30	16.49	<.001
tDCS (fixed dose vs sham)	−0.51 (0.15)	1834	−3.17	.001
tDCS (individualized dose vs sham)	−0.73 (0.15)	1834	−4.56	<.001
Time	−0.29 (0.16)	1835	−1.79	.07
Sex	−0.45 (0.32)	22	−1.40	.17
tDCS (fixed dose vs sham) × time	0.55 (0.23)	1835	2.41	.02
tDCS (individualized dose vs sham) × time	0.91 (0.23)	1835	3.96	<.001
tDCS (fixed dose vs sham) × sex	0.19 (0.22)	1835	0.88	.37
tDCS (individualized dose vs sham) × male sex	0.35 (0.22)	1835	1.58	.11
Time × sex	0.45 (0.23)	1835	1.98	.04
tDCS (fixed dose vs sham) × time × sex	−0.05 (0.32)	1835	−0.17	.86
tDCS (individualized dose vs sham) × time × sex	−0.51 (0.32)	1835	−1.59	.11
**Amplitude change estimate: tDCS type × sex + participant No. + trial No.**				
Fixed effects				
Intercept	28.47 (44.05)	56	0.65	.52
tDCS (fixed dose)	74.99 (56.02)	910	1.34	.18
tDCS (individualized dose)	144.26 (55.74)	908	2.59	.01
Male sex	3.49 (61.74)	56	0.06	.96
tDCS (fixed dose) × male sex	−119.54 (78.3)	903	−1.53	.13
tDCS (individualized dose) × male sex	−49.78 (78.89)	904	−0.63	.53

Abbreviations: LME, linear mixed effects; MEP, motor-evoked potential; tDCS, transcranial direct current stimulation.

^a^ Full model including all 3 tDCS conditions (fixed dose, individualized dose, and sham) and interaction terms.

^b^ Direct comparison between fixed-dose and individual-dose tDCS.

^c^ Models evaluating percentage change with tDCS-by-sex interactions.

**Figure 2.**
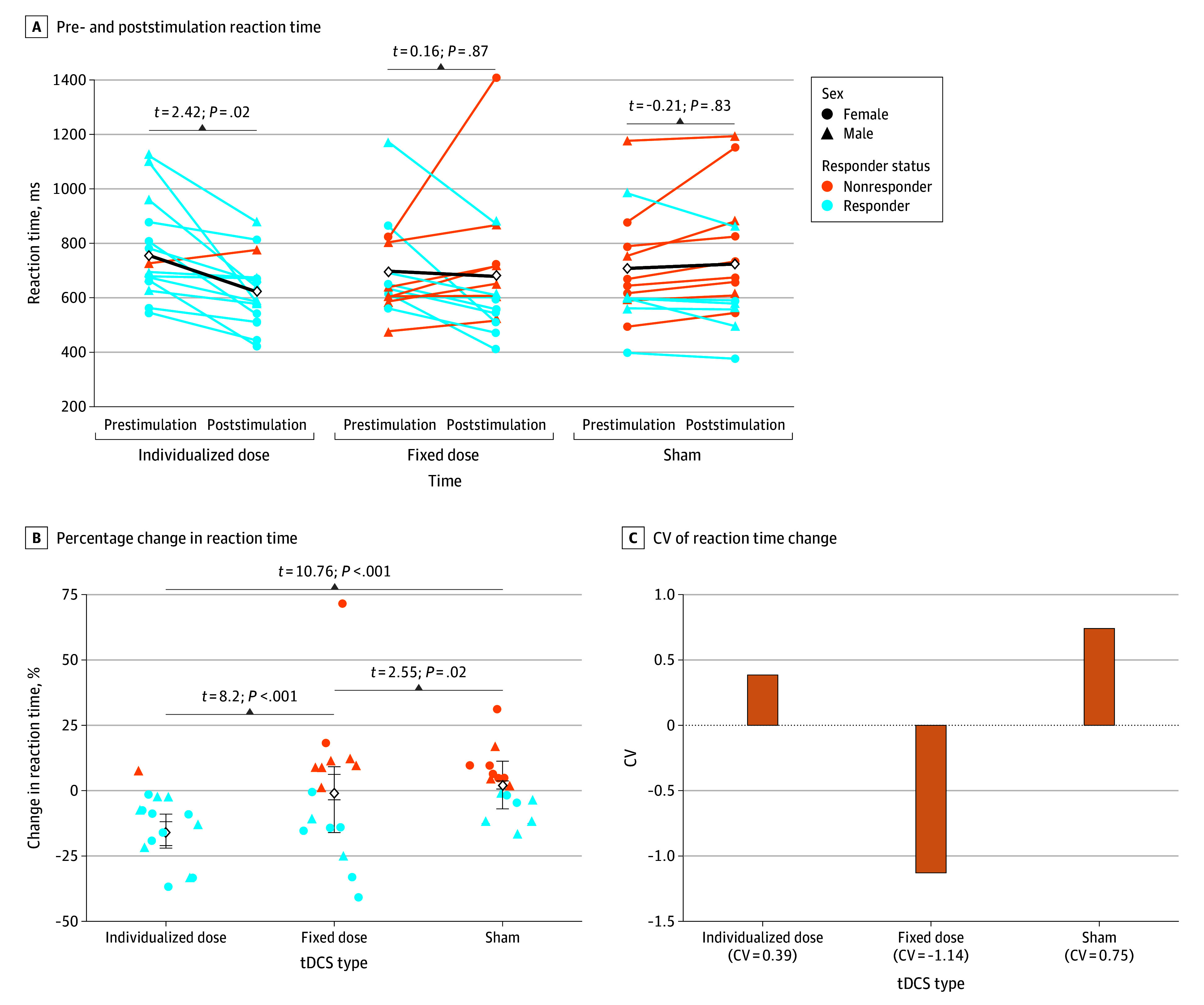
Associations of Fixed- and Individualized-Dose Transcranial Direct Current Stimulation (tDCS) With Behavior Outcomes (Reaction Time) The *t* tests were 2-tailed. CV indicates coefficient of variation.

Estimated marginal means indicated that individualized-dose stimulation was associated with the largest reaction time reduction (estimated marginal mean [SD]: before, 753.0 [41.1] ms; after 619.0 [41.1] ms; change [Δ] = 133.6 ms; SE, 10.2 ms; *z* score ratio, 13.09; *P* < .001), whereas fixed-dose stimulation was not associated with a reduction (estimated marginal mean [SD]: before, 694.0 [41.1] ms; after, 680.0 [41.1] ms; Δ = 14.6 ms; SE, 10.1 ms, *z* score ratio, 1.45; *P* = .70) (Figure 2A). Reaction time was higher with sham stimulation, but the difference was not statistically significant (estimated marginal mean [SD]: before, 708.0 [41.1] ms; after, 724.0 [41.1] ms; Δ = −16.3 ms; SE, 10.2 ms; *z* score ratio, −1.59; *P* = .60). Poststimulation reaction times were significantly faster in the individualized-dose condition compared with the fixed-dose condition (individualized, 619.0 ms; fixed, 680.0 ms; Δ = 60.7 ms; SE, 10.1 ms; *z* score ratio, 6.02; *P* < .001).

#### Sex Differences in Reaction Time

Individualized-dose tDCS was associated with statistically significant reaction time improvements in both sexes of 113.8 ms in women (estimated marginal mean [SD]: before, 682.0 [57.2] ms; after, 568.2 [57.2] ms; *z* = 7.89; *P* < .001) and 153.4 ms in men (estimated marginal mean [SD]: before, 823.4 [57.2] ms; after: 670.0 [57.2] ms; *z* = 10.63; *P* < .001). In contrast, fixed-dose stimulation was associated with in a significant reaction time decrease in women (before, 695.5 ms; after, 637.5 ms; Δ = 58.0 ms; *z* = 2.93; *P* = .003) but not in men (before, 693.5 ms; after, 685.9 ms; Δ = 7.6 ms; *z* = 0.20; *P* = .84). Notably, reaction time after sham stimulation was lower by 21.7 ms (from 677.5 to 655.8 ms) in women (*P* = .93) and 7.6 ms in men (from 693.5 to 685.9 ms), but these differences were not statistically significant (*P* > .99). These findings align with a significant 3-way interaction (tDCS fixed dose × time × sex: β [SE], 97.55 [28.68]; *P* < .001), underscoring a sex-dependent modulation of tDCS outcomes and the larger reductions in reaction time associated with individualized dosing, especially in men.

#### Subgroup Analysis Between Fixed-Dose and Individualized-Dose tDCS

The LME analysis revealed a significant tDCS type–by–time interaction (β [SE], −91.78 [20.74] ms; *t*_3179_ = −4.43; *P* < .001), indicating a greater reaction time reduction with individualized stimulation (Table). A significant tDCS type–by–sex interaction (β [SE], 87.69 [20.98] ms; *t*_3179_ = 4.17; *P* < .001) and a trend-level 3-way interaction (β [SE], −54.06 [29.50] ms; *t*_3179_ = −1.83; *P* = .07) suggested sex-dependent outcomes. Post hoc tests confirmed that reaction times after individualized-dose stimulation (estimated marginal mean [SD], 619.0 [36.2] ms) was significantly faster than before stimulation (estimated marginal mean [SD], 753.0 [36.2] ms; Δ = 133.6 ms; SE, 10.5 ms; *z* score, 12.73; *P* < .001), fixed dose before stimulation (694.0 ms; Δ = 75.3 ms; SE, 10.5 ms; *z* score, 7.17; *P* < .001), and fixed dose after stimulation (680.0 ms; Δ = 60.5 ms; SE, 10.4 ms; *z* score, 5.83; *P* < .001). Stratified analyses showed that individualized-dose tDCS was associated with larger changes than fixed-dose tDCS for reaction times in both women (before, 512.3 ms; after, 480.9 ms; Δ = 31.4 ms; SE, 10.4 ms; *z* score, 3.03; *P* = .01) and men (before, 498.7 ms; after, 469.4 ms; Δ = 29.3 ms; SE, 10.5 ms; *z* score, −2.79; *P* = .03).

#### Proportional Change in Reaction Time From Baseline Performance

The LME that was used to examine percentage change in reaction time, with tDCS type, sex, and their interaction as fixed effects, and participant and trial numbers as random factors is shown in Table. The model revealed significant main effect sizes for both active (fixed- and individualized-dose) stimulation conditions. Compared with sham, fixed-dose tDCS was associated with a reduction in reaction time (β [SE], −11.43% [2.28%]; *t*_2374_ = −5.01; *P* < .001), and individualized-dose tDCS was associated with a greater reduction (β [SE], −23.55% [2.28%]; *t*_2374_ = −10.32; *P* < .001). Significant interactions were observed between tDCS type and sex for which the fixed dose–by–male sex interaction (β [SE], 14.61% [3.23%]; *t*_2374_ = 4.52; *P* < .001) and the individualized dose–by–male sex interaction (β [SE], 12.36% [3.23%]; *t*_2374_ = 3.83; *P* < .001) indicated attenuated reaction time improvement in men.

Estimated marginal means (Figure 2B) showed the greatest improvement with individualized-dose tDCS (before, 76.75%; after, 90.00%; Δ = −12.17%; SE, 3.61%), followed by fixed-dose (before, 75.00%; after, 76.07%; Δ = 1.07%; SE, 3.61%), and sham (before, 79.00%; after, 84.20%; Δ = 5.20%; SE, 3.61%). Post hoc Tukey-adjusted pairwise comparisons supported these findings in which the individualized dose outperformed the fixed dose (Δ = −13.25%; SE, 1.62; *t*_94_ = −8.20; *P* < .001) and sham (Δ = −17.37%; SE, 1.61%; *t*_94_ = −10.76; *P* < .001), while the fixed dose also showed modest benefits over sham (Δ = −4.13%; SE, 1.61%; *t*_94_ = −2.55; *P* = .02).

To assess interindividual variability in tDCS response, standardized coefficients of variation (CVs) were computed for percentage reaction time change (Figure 2C). Individualized-dose stimulation showed the lowest standardized CV (0.39), indicating the most consistent responses, followed by sham (0.75). Fixed-dose tDCS exhibited the highest standardized CV (−1.14), reflecting the greatest variability.

#### Association Between Current Density and Reaction Time

**Figure 3.**
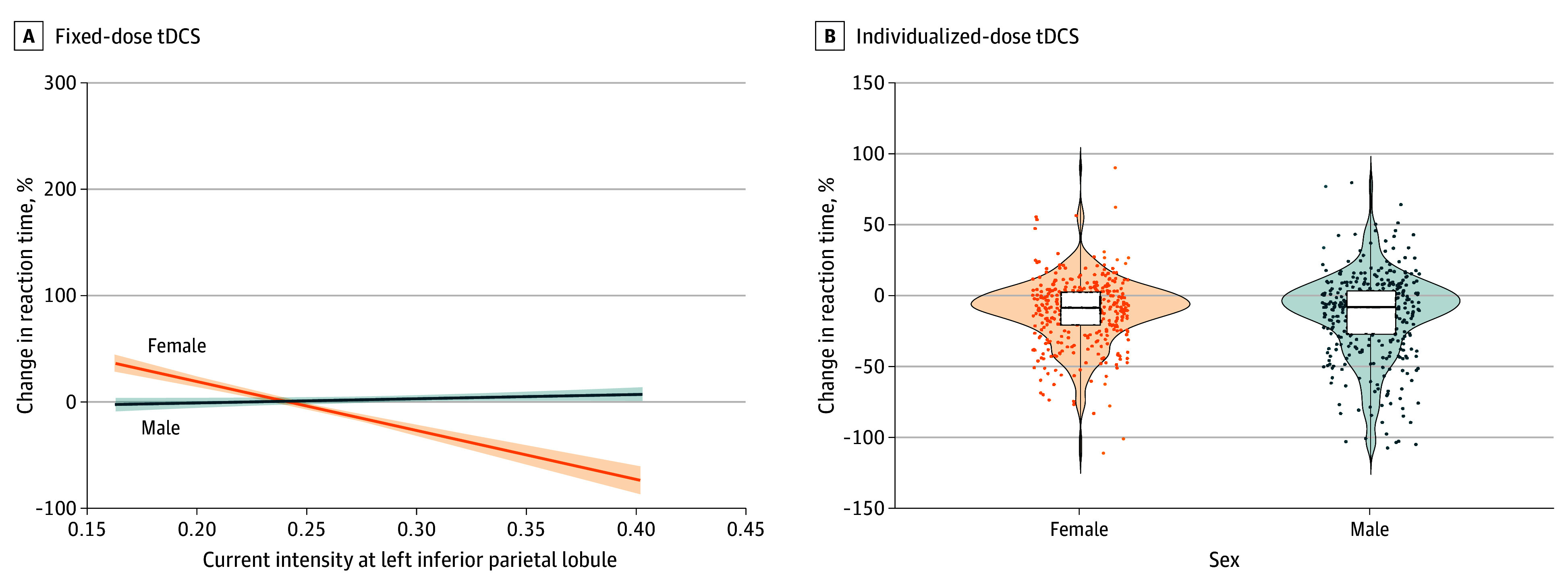
Violin Plot of the Association Between Current Intensity at the Left Inferior Parietal Lobule and Percentage Change in Reaction Time Across Sexes A, Shading indicates the SD. B, The horizontal line inside the box indicates the median; the top of the box, the third quartile, and the bottom of the box, the first quartile. The vertical line from top to bottom is the maximum to minimum quartiles. The shading indicates the estimated probability density function of the data distribution. Each dot representes data from an individual trial.

In the fixed tDCS condition, percentage reaction time change was significantly associated with current density at the target region, by sex, and their interaction. The model showed an association between current density and percentage reaction time change (β [SE], −460.64 [44.39]; *t*_792_ = −10.38; *P* < .001), with men showing attenuated effects for sex (β [SE], −121.13 [13.37]; *t*_792_ = −9.06; *P* < .001) and interaction (β [SE], 502.43 [51.53]; *t*_792_ = 9.75; *P* < .001). Estimated marginal means at a current density of 0.26 indicated a greater reaction time reduction in women (before, 75.82%; after, 68.39%; Δ = −7.43%; SE, 1.95%) compared with men (before, 78.04%; after, 80.61%; Δ = 2.57%; SE, 1.89%) (Figure 3A). In the individualized-dose tDCS condition, sex was not associated with percentage reaction time change (β [SE], 1.70 [2.27]; *t*_796_ = 0.75; *P* = .45), indicating similar outcomes in men and women (Figure 3B). The estimated marginal means were −13.0% (SE, 1.61%) for women and −11.3% (SE, 1.61%) for men.

### Association of tDCS With MEP Amplitude

#### Overall Model

**Figure 4.**
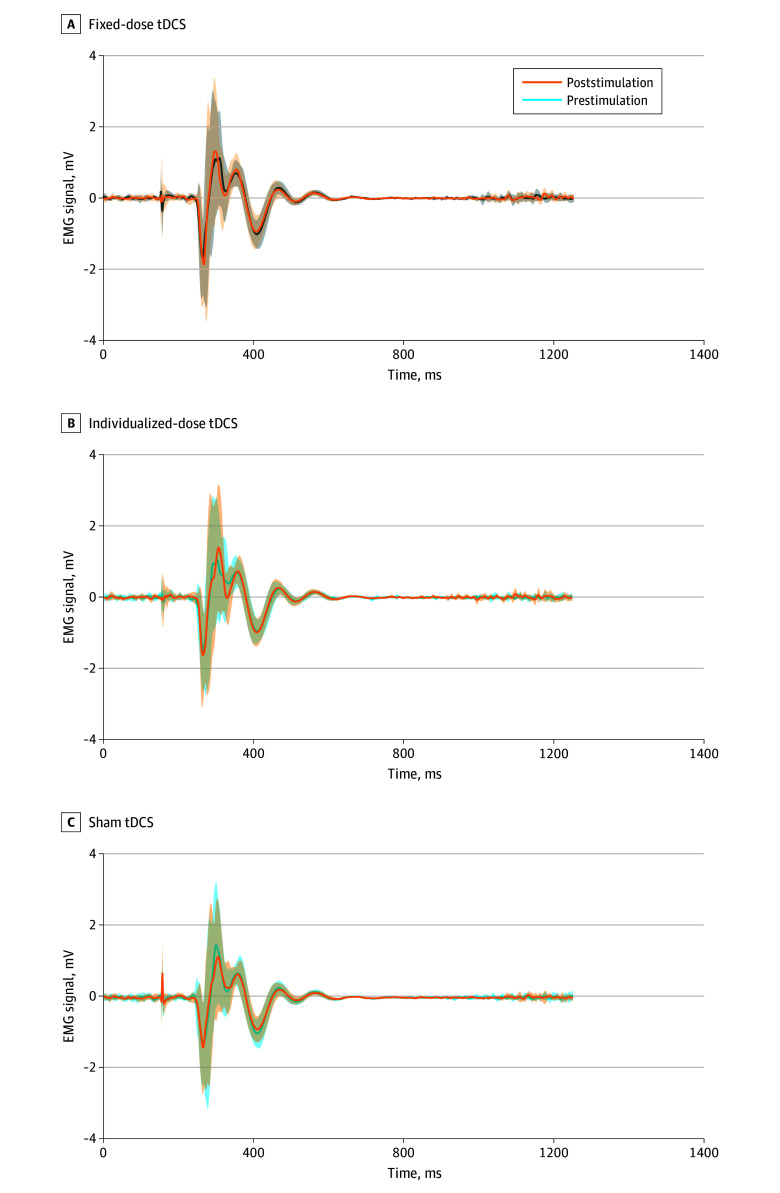
Association of Transcranial Direct Current Stimulation (tDCS) With Neurophysiologic Outcomes (Motor-Evoked Potential Amplitude) The shaded regions indicate the standard error of the mean. EMS indicates electromyography.

Grand average EMG traces before and after stimulation across tDCS conditions showed the greatest MEP increase after individualized-dose stimulation, followed by fixed-dose stimulation, with no change in sham (Figure 4). In the overall model, LME analysis revealed a significant main effect size of stimulation condition on MEP amplitude in fixed-dose tDCS (β [SE], −0.51 [0.15]; *t*_1834_ = −3.17; *P* = .001) and individualized-dose tDCS (β [SE], −0.73 [0.15]; *t*_1834_ = −4.56; *P* < .001), with significant interactions between stimulation type and time for fixed-dose tDCS (β [SE], 0.55 [0.23]; *t*_1835_ = 2.41; *P* = .02) and individualized-dose tDCS (β [SE], 0.91 [0.23]; *t*_1835_ = 3.96; *P* < .001), indicating differential poststimulation outcomes by condition (Table). A small, but significant time-by-sex interaction (β [SE], 0.45 [0.23]; *t*_1835_ = 1.98; *P* = .04) was also observed. No significant 3-way interactions emerged.

Estimated marginal means showed no amplitude change in the sham condition (before, 3.89; after, 3.82; Δ = −0.07; SE, 0.11; *t*_796_ = −0.61; *P* = .82; Cohen *d* = −0.06) (eFigure, A in Supplement 1). In contrast, the amplitude increased under fixed-dose stimulation (before, 3.48; after, 3.94; Δ = 0.46; SE, 0.11, *t*_796_ = 4.12; *P* < .001; Cohen *d* = 0.40) and individualized-dose stimulation (before, 3.34; after, 3.92; Δ = 0.58; SE, 0.11; *t*_796_ = 5.09; *P* < .001; Cohen *d* = 0.51). The difference between amplitudes for individualized and fixed doses after stimulation was small and not statistically significant.

#### Proportional Change in MEP From Baseline Performance

An LME was fitted to examine the associations of tDCS condition and sex with percentage change in MEP amplitude (Table), with random intercepts for participant and trial numbers. The model revealed a significant main effect of individualized-dose tDCS compared with sham (β [SE], 144.26 [55.74]; *t*_908_ = 2.59; *P* = .01). In contrast, fixed-dose tDCS did not show a statistically significant difference from sham (β [SE], 74.99 [56.02]; *t*_910_ = 1.34; *P* = .18).

Post hoc pairwise comparisons (Tukey-adjusted) of (1) individualized dose vs sham yielded a mean difference of 84.5% (SE, 39.2%; *t*_905_ = −2.16; *P* = .04), (2) fixed dose vs sham showed a difference of 50.1% (SE, 39.5%; *t*_906_ = −1.27; *P* = .41), and (3) individualized vs fixed dose differed by 34.4% (SE, 38.7%; *t*_899_ = 0.89; *P* = .64) (eFigure, B in Supplement 1). Standardized CV analysis revealed that individualized-dose tDCS had the lowest variability in amplitude response (−0.79), followed by sham (−0.32), while fixed-dose tDCS showed the highest variability (1.12).

### Conversion of Nonresponders to Responders

Among the 16 participants who underwent both fixed-dose and individualized-dose tDCS sessions, 15 responded under individualized dosing, whereas only 9 (in terms of reaction time) responded under the fixed-dose protocol. Notably, 7 participants (participants 1 [male], 3 [female], 5 [male], 8 [female], 12 [male], 13 [male], and 9 [male]) were nonresponders in the fixed-dose condition, of whom all except participant 9 became responders following individualized-dose stimulation. Of these individuals, participants 1, 5, 8, and 9 received an increased dose of 3 mA, whereas participants 3, 12, and 13 received a reduced dose of 1 mA.

In total, 11 participants (participants 1 [male], 2 [male], 3 [female], 5 [male], 6 [female], 8 [female], 9 [male], 10 [male], 11 [female], 12 [male], and 13 [male]) received individualized doses different from the standard 2-mA fixed dose. The remaining 5 participants (participants 4 [female], 7 [female], 14 [female], 15 [female], and 16 [male]), retained the fixed 2-mA dose, even during the individualized-dose condition. Participant 9 was the only individual unresponsive to both stimulation protocols (across both reaction time and MEP measures). However, there was no correlation between percentage reaction time change and percentage amplitude change in MEP. The subgroup findings underscore the importance of personalized dose titration, especially in converting nonresponders to the fixed dose to responders.

### Test-Retest Reliability

The ICC for test-retest reliability for reaction time was 0.583 (95% CI, 0.427-0.772), indicating moderate reliability^38^ (*F*_15,520_ = 86.8; *P* < .001). This finding suggests that reaction time was consistent in repeated trials across sham sessions. In contrast, MEP amplitude showed lower reliability (ICC, 0.140 95% CI, 0.059-0.324; *F*_14,280_ = 4.5; *P* < .001), consistent with known physiologic variability in TMS responses.^39,40^

To rule out systematic effects of session day, additional LME models included session day as a covariate. No significant main effect sizes or interactions with tDCS condition were observed for reaction time or MEP amplitude.

The post hoc power analysis revealed that the LME model had 89% power to detect the fixed effect of tDCS type on reaction time and 86% power to detect the time–by–tDCS type interaction. For MEP amplitude, the power to detect the main effect of tDCS was 80%, while the interaction effect yielded 76% power. These values emphasize that the study design had sufficient sensitivity to detect medium to large effects.

## Discussion

This comparative effectiveness study suggests that individualized tDCS is associated with greater improvements in behavioral performance and cortical exictability than a fixed-dose approach. Personalized dosing was associated with a greater reaction time reduction of 13.25% compared with fixed dosing, significantly lower variability (−1.14 vs 0.39), minimal sex differences, and conversion of nonresponders to responders, supporting previous findings on dose optimization.^6,41^ Additionally, individualized-dose stimulation had the largest proportional change in MEP from baseline performance when compared with fixed-dose and sham conditions, reinforcing concerns about fixed-dose limitations.^41^ These results highlight the consistent benefit of individualized-dose stimulation with stable behavior outcomes and underscore the need for tailored stimulation protocols to maximize tDCS efficacy and reliability, particularly as tDCS moves toward home-based applications.^42^

Although dose titration proved beneficial, 1 participant did not respond to either fixed- or individualized-dose stimulation. While the precise reason for this lack of observable response remains unclear, previous research suggests that trait-related factors may modulate stimulation effects,^3^ highlighting the need for further investigation.^43^

### Limitations

This study has some limitations. The small sample size, although powered for within-participant effects, may limit generalizability and warrants replication in larger cohorts. Additionally, subgroup analyses, particularly sex-stratified outcomes, should be interpreted cautiously due to the limited sample size. Only 1 montage was tested, while other configurations (eg, frontal for depression, motor for rehabilitation) warrant investigation. The MEPs assessed cortical excitability, but real-time tDCS and functional MRI might provide stronger neurophysiologic evidence. Additionally, all MRIs were simulated with default conductivity values for bone, white matter, gray matter, and cerebrospinal fluid (since individual estimates for these values were not performed). We understand that such default assumptions deter the precision, especially when personalized protocols are designed. Future models with values estimated for each individual may provide precise estimates of electric field intensity in the target region. Finally, using a standard Montreal Neurological Institute template for dose individualization may have limited precision, and population-specific templates may have provided precise improvement in accuracy.

Although increasing the total dose may improve targeting, it risks stimulating off-target regions, such as the motor cortex, potentially affecting reaction time, making the conventional montage, which covers a broader area, advantageous in this context. Multichannel tDCS, a more focal stimulation technique, enhances treatment efficacy.^43,44,45,46^ It offers an opportunity to individualize the montage, but precise electrode placement is crucial, especially for home use. Future research should optimize multichannel tDCS by tailoring both montage and dose.

## Conclusions

Overall, this comparative effectiveness study provides compelling experimental evidence that individualized tDCS is associated with improved behavioral and neurophysiologic outcomes compared with a fixed-dose neuromodulation strategy; individualized dosing also had lower variability than fixed-dose or sham approaches. The consistent outcomes across participants, minimal sex-related variability, and improved outcomes in nonresponders who received individualized dosing suggests that it may have translational potential, especially for home-based and precision medicine applications.

## Notes

Supplement 1. **eFigure.** Before and After Amplitudes and Percent Amplitude Changes Across Participants and tDCS Conditions

Supplement 2.
Data Sharing Statement
