# Supplementary material for: Personalized Transcranial Direct Current Stimulation for Behavioral and Neurophysiologic Outcomes
Source: JAMA Netw Open. 2025 Aug 25;8(8):e2526148. doi: 10.1001/jamanetworkopen.2025.26148 (PMC12379099; doi:10.1001/jamanetworkopen.2025.26148)
Supplement: Supplement 1. — eFigure. Before and After Amplitudes and Percent Amplitude Changes Across Participants and tDCS Conditions [file jamanetwopen-e2526148-s001.pdf]

Supplemental Online Content

Bhattacharjee S, Sivakumar PT, Venkatasubramanian G, et al. Personalized transcranial direct current stimulation for behavioral and neurophysiologic outcomes. *JAMA Netw Open*. 2025;8(8):e2526148. doi:10.1001/jamanetworkopen.2025.26148

eFigure. Before and After Amplitudes and Percent Amplitude Changes Across Participants and tDCS Conditions

This supplemental material has been provided by the authors to give readers additional information about their work.

© 2025 Bhattacharjee S et al. *JAMA Network Open*.

eFigure. Before and After Amplitudes and Percent Amplitude Changes Across Participants and tDCS Conditions

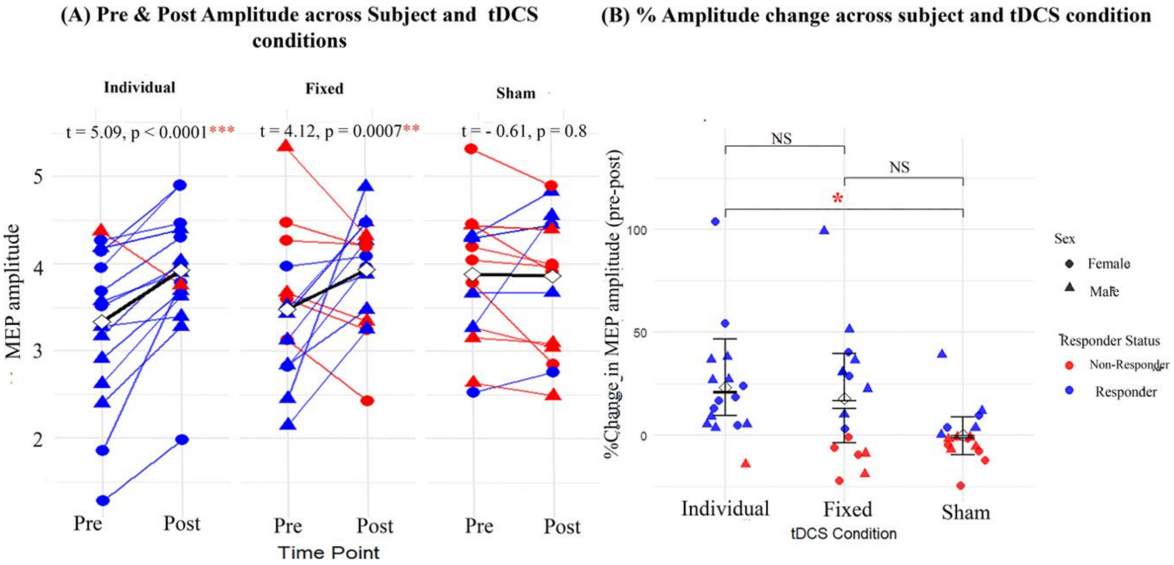

Figure 1S

(A) Pre- and post-stimulation MEP amplitude by subject for each tDCS condition. Individualized stimulation resulted in a significant increase (t = 5.09; *P* < .0001), as did fixed stimulation (t = 4.12; *P* = .0007), whereas sham showed no significant change (t = –0.61; *P* = .80). (B) Percent change in MEP amplitude across tDCS conditions by subject, stratified by sex and responder status. Individualized tDCS showed the greatest average increase in amplitude, with reduced variability across participants. Although the differences between conditions were not statistically significant in direct comparisons, a numerical advantage was observed for individualized stimulation.

© 2025 Bhattacharjee S et al. *JAMA Network Open*.
