# Supplementary material for: Personalized Transcranial Direct Current Stimulation for Behavioral and Neurophysiologic Outcomes
Source: JAMA Netw Open. 2025 Aug 25;8(8):e2526148. doi: 10.1001/jamanetworkopen.2025.26148 (PMC12379099; doi:10.1001/jamanetworkopen.2025.26148)
Supplement: Supplement 2. — Data Sharing Statement [file jamanetwopen-e2526148-s002.pdf]

Data Sharing Statement

Bhattacharjee. Personalized Transcranial Direct Current Stimulation for Behavioral and Neurophysiologic Outcomes. *JAMA Netw Open*. Published August 25, 2025. doi:10.1001/jamanetworkopen.2025.26148

Data
Data available: Yes
Data types: Deidentified participant data
How to access data: Data will be made available upon reasonable request to the corresponding author bhattacharya.sagarika7@gmail.com
When available: With publication

Supporting Documents
Document types: None

Additional Information
Who can access the data: Researchers whose proposed use of the data has been approved
Types of analyses: Any analysis with justification.
Mechanisms of data availability: with a signed data access agreement.
